# Developmental Toxicity and Cardiotoxicity of N, N-Dimethylaniline in Zebrafish Embryos

**DOI:** 10.3390/toxics13020125

**Published:** 2025-02-08

**Authors:** Bin Liu, Bo Peng, Yan Jin, Yijie Tao, Wenping Xu, Yang Zhang, Zhong Li

**Affiliations:** 1 Shanghai Key Laboratory of Chemical Biology, School of Pharmacy, East China University of Science and Technology, Shanghai 200237, China; liubin0157@sina.com (B.L.); y20230115@mail.ecust.edu.cn (B.P.); tyj57836608@163.com (Y.T.); xuwp@ecust.edu.cn (W.X.); 2 Qingpu District Agro-Technology Extension Service Center, Shanghai 201799, China; jinyannj998@163.com

**Keywords:** N, N-dimethylaniline, developmental toxicity, cardiotoxicity, zebrafish

## Abstract

N, N-Dimethylaniline is an important chemical intermediate and an important metabolite of the pesticide Fenaminosulf. It is widely used in chemical production, but there is an extreme paucity of environmental risk assessments for N, N-dimethylaniline.: In this study, the cardiotoxicity of continuous exposure to N, N-dimethylaniline (20, 40, and 80 μg/mL) for 72 h was evaluated using zebrafish embryos.: The study found that N, N-dimethylaniline not only exhibits developmental toxicity to zebrafish embryos, leading to abnormalities such as pericardial edema, yolk sac edema, and spinal curvature, but also induces oxidative stress, lipid accumulation, and apoptosis, particularly affecting the heart region. Cardiac function indicators such as pericardial area, sinus venosus (SV) and bulbar artery (BA) distance, heart rate, and red blood cell (RBC) rate were all significantly altered due to exposure to N, N-dimethylaniline, with impaired cardiac morphology and structure and the downregulation of gene expression related to heart development and function (*myl7*, *vmhc*, *myh6*, *bmp4*, *tbx2b*, and *has2*).: The research findings suggest that the heart may be the potential target organ for the toxic effects of N, N-dimethylaniline, providing a scientific basis for the rational use of this compound and environmental protection. Furthermore, it enhances public awareness of the safety of substances that may degrade to produce N, N-dimethylaniline during their use.

## 1. Introduction

*Zizania latifolia* (*Z. latifolia*, Turcz, fam. Poaceae), native to East Asian countries, has a cultivation history spanning 2000 years [1]. *Zizania latifolia*, the second most grown aquatic vegetable in China, covers more than 70,000 hectares, with an annual production value of RMB 3 billion [2]. Fenaminosulf is a moderately toxic, protective, and systemic penetrating fungicide that is widely used in China for *Zizania latifolia* cultivation and is effective against a broad spectrum of fungal diseases. Proper use of Fenaminosulf not only controls diseases in *Zizania latifolia* fields but also promotes *Zizania latifolia* harvests [3]. Research indicates that an aqueous solution of Fenaminosulf is unstable in the light, and its decomposition is accelerated by light, heat, and alkaline substances. Fenaminosulf rapidly degrades in aquatic environments, with a half-life ranging from 2.12 to 3.53 h, and its primary metabolite is N,N-dimethylaniline [4]. N, N-Dimethylaniline is a chemical intermediate used in the synthesis of dyes, and it also serves as a solvent and an auxiliary agent for methylation processes [5]. Aromatic amines have long been recognized as potential carcinogens, with N, N-dimethylaniline being of concern due to its widespread use in industry. Although previous studies have indicated that N, N-dimethylaniline possesses certain carcinogenicity and genotoxicity [5,6], there is still a lack of comprehensive assessment of its potential risk in the aquatic environment, where its persistence and potential bioaccumulation may cause unknown long-term effects on aquatic ecosystems. Therefore, a risk assessment of N, N-dimethylaniline in the aquatic environment is particularly urgent, which will not only help to protect the health of aquatic organisms but also provide a scientific basis for the development of environmental protection policies.

During the embryonic development of vertebrates, the heart is the first organ to form and function, primarily responsible for supplying blood to all parts of the body and maintaining blood circulation [7]. Cardiovascular diseases have consistently been the leading cause of death globally, with one-third of global deaths in 2021 attributed to cardiovascular conditions [8]. Exposure to chemical pollutants increases the risk of cardiovascular disease [9]. For example, exposure to arsenic, lead, cadmium, and copper increases the risk of cardiovascular disease and coronary heart disease [10]. Chimney sweeps occupationally exposed to PAHs have elevated serum levels of homocysteine and cholesterol and a higher risk of cardiovascular disease [11]. Exposure to household insecticides, especially household insect repellents, increases the risk of cardiovascular disease death in older adults [12]. The cardiotoxicity of chemical pollutants is therefore a real environmental health problem for living organisms.

Zebrafish is a widely recognized model organism in toxicological studies, with the advantages of small size, in vitro development of embryos, a short reproductive cycle, transparent embryos, the formation of all major body systems within 72 h post-fertilization, and a high degree of homology with the human genome [13,14,15]. Zebrafish are sensitive to environmental pollutants, so their relevant toxicological data can be reflected in human health as an indicator for environmental risk assessment [16,17,18]. In addition, the early developmental mechanisms of zebrafish are consistent with those of mammals, and the zebrafish model has also been considered the best system for assessing the cardiotoxic effects of drugs due to the transparency of the heart and the simplicity of its structure, which makes it easy to visualize [19,20,21]. For example, Ethoprophos exposure inhibits the production of antioxidant enzymes and activates reactive oxygen species to block the Wnt signaling pathway, inducing developmental defects in cardiac function [22]. Molecular docking results suggest that the cardiotoxicity of Fenpropathrin may be associated with the blockage of voltage-gated ion channels that maintain the electrical potential balance of the cell membrane [23]. Exposure to Isoflucypram causes severe developmental abnormalities in zebrafish embryos, including pericardial edema, yolk sac edema, and coagulation agglutination, as well as delayed hatching and a reduced heart rate. Additionally, the expression of cardiac-specific genes and erythropoiesis-related genes are disrupted [24].

This study aims to systematically investigate the cardiotoxicity of N, N-dimethylaniline in zebrafish embryos, focusing on specific toxicological endpoints such as oxidative stress, gene expression, cardiac function, and morphological changes. By examining these endpoints, the study provides a scientific basis for the rational use of N, N-dimethylaniline and environmental protection. Additionally, the study contributes to enhancing public awareness of the safety of substances like Fenaminosulf, which degrade to N, N-dimethylaniline during use.

## 2. Materials and Methods

### 2.1. Chemicals and Reagents

N, N-Dimethylaniline (≥99.5%, CAS: 121-69-7) and DMSO (analytically pure, ≥99.5%) was purchased from Macklin Biochemical Co., Ltd (Shanghai, China). The stock solution of N, N-dimethylaniline (100 mg/mL) was prepared with DMSO. The working solution for the experimental group was prepared by diluting the stock solution into the embryo culture solution. The working solution for the control group was prepared by diluting DMSO directly into the embryo culture medium, and the final concentration of DMSO was the same as that in the highest-concentration working solution (<0.1%) [25].

### 2.2. Zebrafish Rearing and Embryo Acquisition

Adult wild-type zebrafish and adult transgenic type zebrafish (*Tg (myl7: GFP)*) were purchased from the China Zebrafish Resource Center (Wuhan, China). Zebrafish were reared in the standard culture system at 28.5 °C, pH = 7.2, with a sunshine duration of 14 h, and fed twice daily with brine shrimp. Prior to embryo acquisition, adult zebrafish need to be placed in a spawning tank in a 1:1 male-to-female ratio and kept away from light. At the end of the next morning, to avoid light, zebrafish began to mate, and fertilized eggs were collected after 1 h of mating and placed in a constant temperature incubator at 28.5 °C.

### 2.3. Drug Exposure Experiments on Embryos

The toxicity experiments were performed on zebrafish embryos according to OECD Guideline No. 236 (FET test) [26]. The pre-experiment was set up with a concentration gradient of 0 to 160 μg/mL of a N, N-dimethylaniline working solution, and 20, 40, and 80 μg/mL drug concentrations were selected for the experimental study based on the mortality rate. At 6 h post-fertilization (hpf), 30 zebrafish embryos were individually exposed to 5 mL of the corresponding N, N-dimethylaniline working solution in a 6-well culture plate. All embryos were cultured in a standard incubator during the experiment. The working solution was changed every 24 h. Before each solution change, we washed the containers with deionized water to remove possible residues of the old solution and other impurities. The total time of the drug exposure experiment was 72 h, and the test was performed in 3 replications. The development of embryos was observed using a stereomicroscope (Z7450T, Nikon, Shanghai, China) at the corresponding time period, and the survival and hatching rates were counted. Dead embryos were defined as those without a visible heartbeat and any spontaneous movement. Hatched embryos were defined as those that hatched completely from the egg membrane.

### 2.4. Cardiac Morphologic and Functional Analysis

At the end of the exposure of zebrafish embryos, zebrafish embryo images were captured using a stereomicroscope (Z7450T, Nikon, Shanghai, China), and the heart rate of the embryos was counted. Heart rate was derived by counting the number of heartbeats per minute. The pericardial area and the sinus venosus (SV) and bulbar artery (BA) spacing of the zebrafish embryos were calculated using Image J software (version 1.54f). Three replicate measurements were performed for each embryo and averaged as the final result. The red blood cell (RBC) flow images of the posterior cardinal vein (PCV) of the zebrafish were taken using a stereomicroscope (Z7450T, Nikon, Shanghai, China), and the RBC flow rate was derived by measuring the distance travelled by the RBCs at a given time using Image J software; three replicate measurements were taken for each embryo, and the average value was taken as the final result [27]. The images of transgenic-type zebrafish embryo (*Tg (myl7: GFP)*) heart fluorescence were captured for analysis using a confocal microscope (Nikon Inc., Melville, NY, USA). During imaging, it was ensured that all embryos were in the same position and orientation and that standardized imaging parameters were used, including the same exposure time and gain settings, to ensure consistency of the fluorescence intensity.

### 2.5. Oxidative Stress Analysis

To assess the degree of oxidative stress in the zebrafish embryos, embryos were collected for viability checks at the end of the exposure to ensure that all embryos used for the experiments were alive. The levels of reactive oxygen species (ROS), malondialdehyde (MDA), and glutathione (GSH) and the activities of superoxide dismutase (SOD) and catalase (CAT) were also measured. ROS levels were assayed by exposing the zebrafish embryos to 10 μM DCFH-DA solution (Beyotime Biotechnology, S0033S, Shanghai, China) and incubating them for 30 min away from light. The embryos were washed three times with PBS for 5 min each to remove unbound dye. Fluorescence images were taken at a 488 nm excitation wavelength using a confocal microscope (Nikon Inc., Melville, NY, USA). The heart region of the embryo was selected for analysis using Image J software, and the average fluorescence intensity of the region was calculated. Meanwhile, the MDA and GSH contents and the activities of SOD and CAT were determined by the colorimetric method using kits from Shanghai Sangong Biotech Co., Ltd. (Shanghai, China), and the absorbance was measured by a UV–visible spectrophotometer (Thermo Fisher, Shanghai, China) at specific wavelengths (MDA: 532 nm; GSH: 412 nm; SOD: 560 nm; and CAT: 240 nm) for quantitative analysis. These indices reflect the effects of N, N-dimethylaniline exposure on oxidative stress in zebrafish embryos.

### 2.6. Lipid Accumulation and Apoptosis Analysis

At the end of the exposure, the zebrafish embryos were stained for Oil Red O to assess the degree of lipid accumulation in zebrafish embryos. To stain, zebrafish embryos need to be washed three times with phosphate buffer solution (PBS) and fixed overnight at 4 °C using 4% paraformaldehyde. The embryos were again washed three times with PBS after fixation was completed and incubated with 60% propylene glycol–PBS for 30 min at room temperature, followed by staining in 0.5% Oil Red O staining solution (prepared in 60% propylene glycol–PBS) for 4 h [28]. After staining, the embryos were washed three times using PBS, and images of oil red staining of zebrafish embryos were captured using a stereomicroscope (Z7450T, Nikon, Shanghai, China) and analyzed by Image J software.

At the end of the exposure, the zebrafish embryos were assessed for apoptosis using the acridine orange (AO) reagent. The embryos need to be washed three times with PBS and incubated with 5 μg/mL AO reagent for 30 min in a constant temperature incubator at 28.5 °C, avoiding light [29]. The embryos were washed three times with PBS at the end of incubation, and fluorescence images were captured using a confocal microscope (Nikon Inc., Melville, NY, USA) and analyzed with Image J software.

### 2.7. Histopathological Analysis

Hematoxylin and eosin (H&E) staining for the histopathological analysis of the zebrafish embryos was performed following the end of exposure. The zebrafish embryos were fixed in 4% paraformaldehyde at 4 °C for 24 h, followed by paraffin embedding, sectioning, and H&E staining at a section thickness of 4 μm. Specific staining steps included the standard procedures of dewaxing, hematoxylin staining, differentiation, eosin staining, dehydration, and sealing. Images were acquired using a sectioning scanner (Pannoramic MIDI, 3DHISTECH, Budapest, Hungary).

### 2.8. Gene Expression Analysis

After exposure, we extracted total RNA from 30 zebrafish embryos using the Trizol reagent (Invitrogen, Carlsbad, USA). RNA concentration and purity were determined, and integrity was verified. Subsequently, cDNA synthesis was performed using a Reverse Transcription Kit (Servicebio, Wuhan, China). qRT-PCR analyses were performed using SYBR Green PCR Master Mix (Servicebio, Wuhan, China) with a reaction system of 20 μL. Each sample was replicated three times, *β-actin* was used as an internal reference gene, and the 2^-ΔΔCt^ method was used to calculate the relative expression of the target gene. The primer sequences of the relevant genes (Table 1) were designed and synthesized by Sangon Biotech (Shanghai, China) Co., Ltd.

### 2.9. Statistical Analysis

The data are expressed as mean ± standard deviation of at least three independent replicate experiments. Data were analyzed for graphing using Graph Pad Prism 9.5 software. The least significant difference method and analysis of variance (ANOVA) were used to analyze the differences between treatment groups. Differences between the control group and the treatment groups were tested using the Dunnett test; “*”, “**”, and “***” indicate significance levels of *p* ≤ 0.05, *p* ≤ 0.01 and *p* ≤ 0.001, respectively.

## 3. Results

### 3.1. Developmental Toxicity of N, N-Dimethylaniline in Zebrafish Embryos

**Figure 1:**
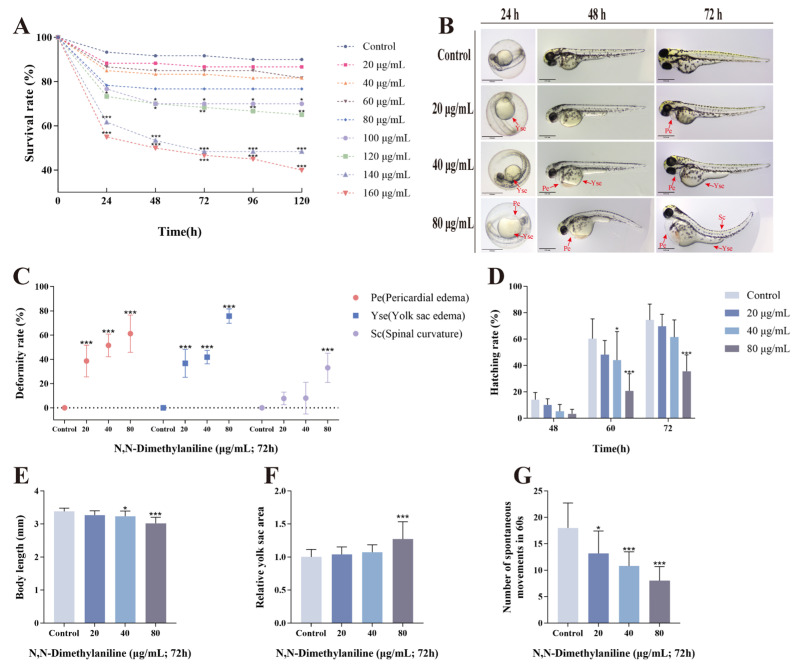
N, N-dimethylaniline induces dysplasia in zebrafish embryos. Survival rate of concentration screening pre-experiments (**A**) at 72 h at each concentration. Schematic diagram of the malformations (**B**) included pericardial edema (Pe), yolk sac edema (Yse), and spinal curvature (Sc). Malformation rate (**C**), hatching rate (**D**), body length (**E**), relative yolk sac area (**F**), and number of spontaneous movements in 60 s (**G**) were also counted. Data are expressed as mean ± standard deviation (mean ± SD), and 30 embryos were analyzed in each experimental and control group *(n* = 3). *p*-values: *** for *p* ≤ 0.001, ** for *p* ≤ 0.01, and * for *p* ≤ 0.05.

To evaluate the developmental toxicity of N, N-dimethylaniline to aquatic organisms, zebrafish embryos were exposed to different concentrations of N, N-dimethylaniline for 120 h at 6 hpf, and the cumulative survival rate was counted for each 24 h period. The results showed that N, N-dimethylaniline caused high mortality of the embryos in a concentration-dependent manner (Figure 1A). Based on the survival rate under the pre-experimental concentrations, we selected 20, 40, and 80 μg/mL as the subsequent experimental concentrations. We observed that the zebrafish embryos treated with N, N-dimethylaniline exhibited pericardial edema (Pe), yolk sac edema (Yse), and spinal curvature (Sc) (Figure 1B,C). Simultaneously, compared to the control group, the hatching of the zebrafish embryos treated with N, N-dimethylaniline was inhibited, accompanied by a reduction in body length and enlargement of the yolk sac (Figure 1D–F). The spontaneous movement of embryos at 24 hpf was also suppressed (Figure 1G). These data indicate that N, N-dimethylaniline possesses developmental toxicity to zebrafish embryos.

### 3.2. N, N-Dimethylaniline Induces Oxidative Stress in Zebrafish Embryos

**Figure 2:**
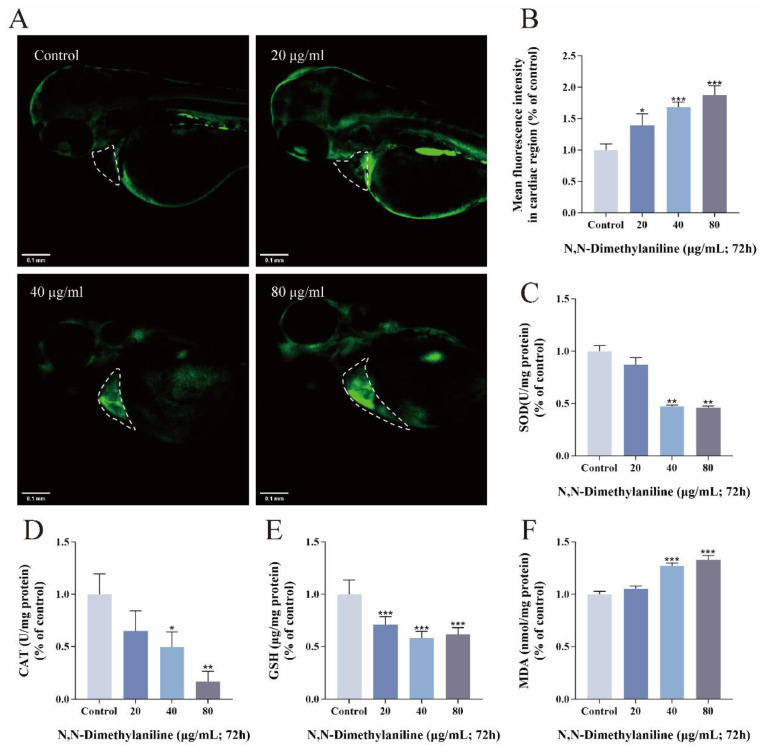
N, N-dimethylaniline induces oxidative stress. Fluorescence images of ROS staining (**A**) and relative fluorescence intensity in the heart region (**B**). SOD activity (**C**), CAT activity (**D**), GSH content (**E**), and MDA content (**F**) at 72hpf. Data are expressed as mean ± standard deviation (mean ± SD), and 30 embryos were analyzed in each experimental and control group *(n* = 3). *p*-values: *** for *p* ≤ 0.001, ** for *p* ≤ 0.01, and * for *p* ≤ 0.05.

Oxidation reduction parameters such as reactive oxygen species (ROS), superoxide dismutase (SOD), catalase (CAT), glutathione (GSH), and malondialdehyde (MDA) can reflect the antioxidant capacity of the zebrafish organism. The intensity of ROS fluorescence in the cardiac region of zebrafish embryos in the treatment group was stronger as the concentration of N, N-dimethylaniline increased compared with the control group (Figure 2A,B). The SOD and CAT activities and GSH level were significantly lower in the treated group than in the control group (Figure 2C–E), and the MDA level was significantly higher than in the control group (Figure 2F). These results suggest that exposure to N, N-dimethylaniline significantly increases the oxidative stress level in zebrafish embryos.

### 3.3. N, N-Dimethylaniline Induces Lipid Accumulation and Apoptosis in Zebrafish Embryos

**Figure 3:**
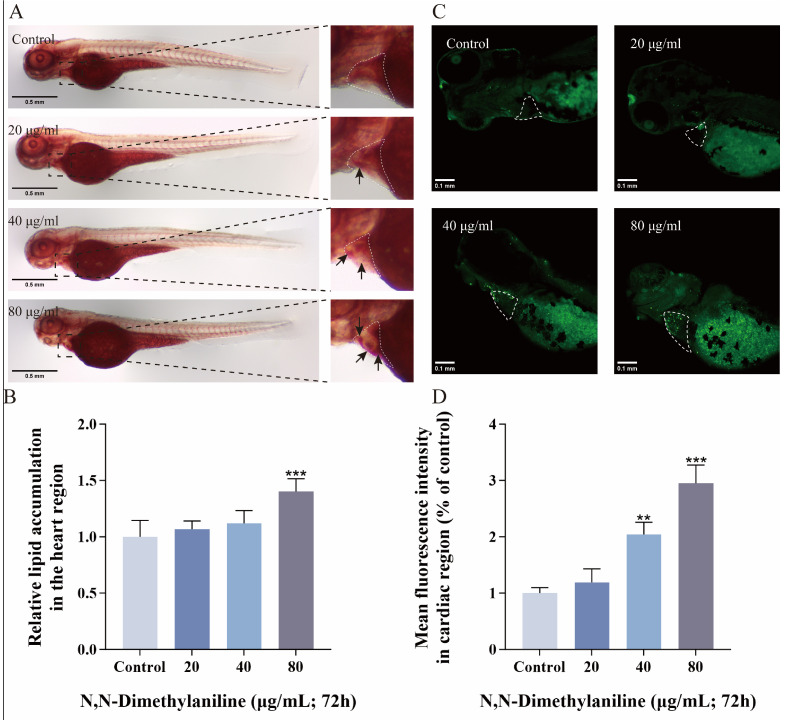
N, N-dimethylaniline induces lipid accumulation and apoptosis. Oil Red O staining images (**A**) and relative degree of lipid accumulation in the heart region (**B**). Apoptotic images stained with AO in the cardiac region at 72 h (**C**) and analyzed for relative fluorescence intensity (**D**). Data are expressed as mean ± standard deviation (mean ± SD), and 30 embryos were analyzed in each experimental and control group *(n* = 3). *p*-values: *** for *p* ≤ 0.001 and ** for *p* ≤ 0.01.

Zebrafish embryos that were continuously exposed for 72 h to different N, N-dimethylaniline concentrations were subjected to oil red staining and AO staining. A qualitative analysis of the images showed an increased degree of lipid accumulation in the cardiac region of the embryos (Figure 3A,B) and an increased number of apoptotic cells in the cardiac region, with a significant increase in fluorescence intensity (Figure 3C,D). These findings indicate that N, N-dimethylaniline can induce apoptosis in the cardiac region of zebrafish embryos and cause abnormal lipid metabolism, leading to lipid accumulation in the cardiac region.

### 3.4. N, N-Dimethylaniline Causes Cardiac Damage in Zebrafish Embryos

**Figure 4:**
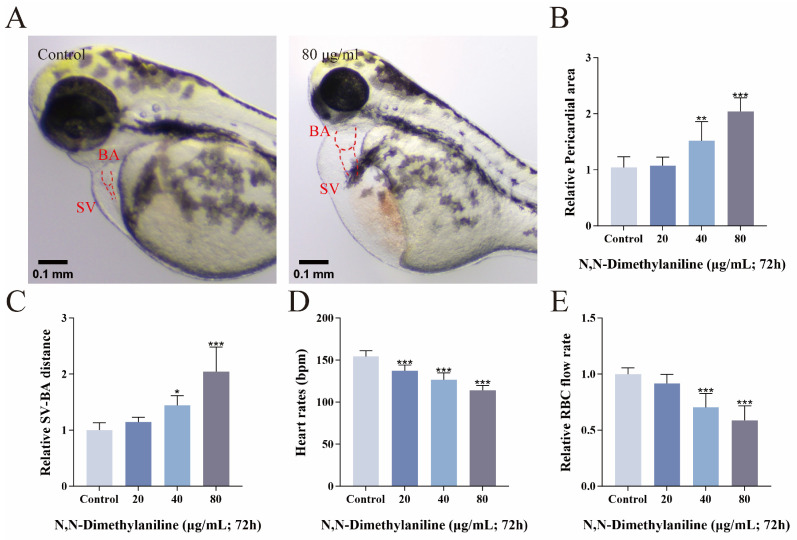
Zebrafish pericardial region images (**A**) and relative pericardial area (**B**). Relative SV-BA distances (**C**), heart rate (**D**), and red blood cell (RBC) flow rate (**E**). Data are expressed as mean ± standard deviation (mean ± SD), and 30 embryos were analyzed in each experimental and control group *(n* = 3). *p*-values: *** for *p* ≤ 0.001, ** for *p* ≤ 0.01, and * for *p* ≤ 0.05.

**Figure 5:**
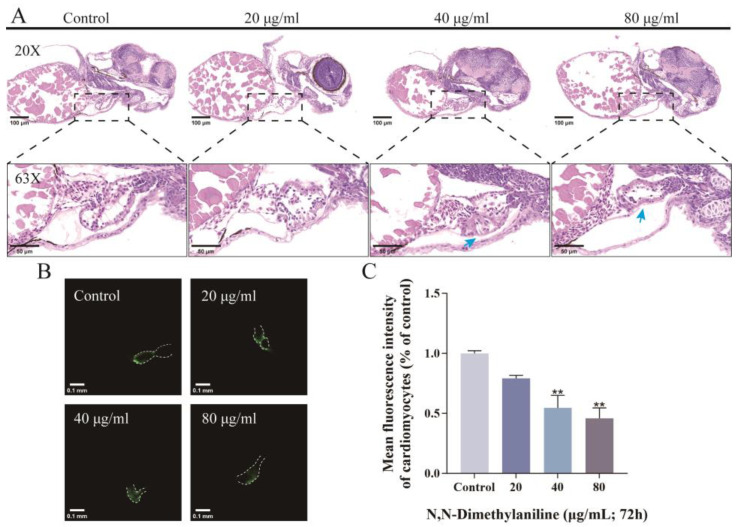
H&E pathology sections (**A**). The blue arrows represent thinning ventricle walls caused by a reduction in cardiac muscle cells. Morphologic change images of cardiac muscle transgenic zebrafish (*Tg (myl7: GFP)*) heart at 72 h (**B**) and cardiac fluorescence intensity statistics (**C**). Data are expressed as mean ± standard deviation (mean ± SD), and 30 embryos were analyzed in each experimental and control group *(n* = 3). *p*-values: ** for *p* ≤ 0.01.

**Figure 6:**
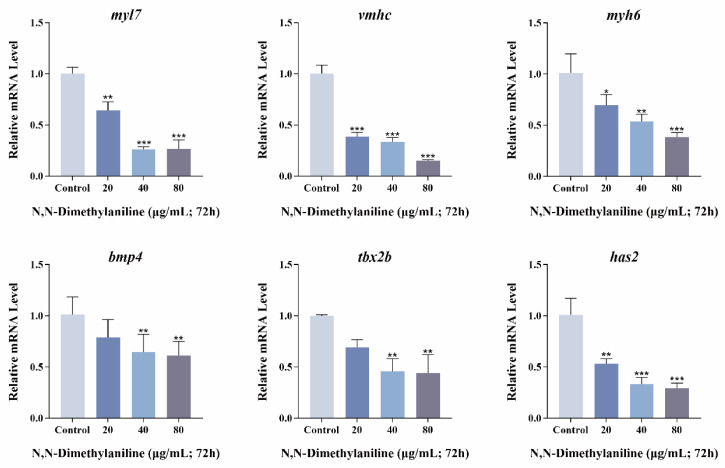
Expression analysis of heart-related genes (*myl7*, *vmhc*, *myh6*, *bmp4*, *tbx2b*, and *has2*) under N, N-dimethylaniline exposure. Data are expressed as mean ± standard deviation (mean ± SD), and 30 embryos were analyzed in each experimental and control group *(n* = 3). *p*-values: *** for *p* ≤ 0.001, ** for *p* ≤ 0.01, and * for *p* ≤ 0.05.

N, N-dimethylaniline affected cardiac morphology and function in zebrafish embryos compared with controls. Following treatment with N, N-dimethylaniline, there was a significant increase in the pericardial area and the distance between SV and BA (Figure 4A–C), as well as inducing a slowing of the heart rate and a significant decrease in the RBC flow rate (Figure 4D,E). Histopathological sections and the fluorescence intensity of cardiac transgenic types showed that N, N-dimethylaniline induced cardiac linearization and damage to cardiomyocytes in zebrafish embryos (Figure 5A–C). In addition, we measured the expression of heart-related genes. The expression levels of genes involved in cardiac development and function, including *myl7*, *vmhc*, *myh6*, *bmp4*, *tbx2b*, and *has2*, were significantly reduced following exposure to N, N-dimethylaniline (Figure 6). In summary, the zebrafish heart may be the potential target for the toxic effects of N, N-dimethylaniline.

## 4. Discussion

N, N-Dimethylaniline, as a key chemical intermediate, has a wide range of applications in the field of chemical production. In addition, it is an important metabolite in the use of pesticides such as Fenaminosulf. However, there is still a lack of in-depth research and understanding regarding the potential toxic effects of N, N-dimethylaniline on the environment, aquatic organisms, and specific organs. In our study, zebrafish embryos were used to assess the toxicity of N, N-dimethylaniline. Our results indicate that N, N-dimethylaniline exhibits developmental toxicity and cardiotoxicity in zebrafish embryos.

In this study, we selected concentrations of 20, 40, and 80 μg/mL of N, N-dimethylaniline for experimentation. These concentrations were determined based on preliminary experimental results and known toxicity data from the literature, encompassing the toxicity thresholds for aquatic organisms, such as the 48 h LC_50_ of 33 mg/L for Oryzias latipes and the 96 h LC_50_ of 78.2 mg/L for Pimephales promelas [30]. Following exposure to N, N-dimethylaniline, the survival rate of zebrafish embryos was significantly adversely affected, accompanied by a series of developmental abnormalities. These abnormalities included hatching inhibition, morphological malformations, reduced body length, and abnormal spontaneous movement behavior. Notably, Pe and Yse were the most significant morphological deformities observed. The effects caused by environmental pollutants on the morphological development of zebrafish embryos have been reported in previous studies [31,32,33].

Changes in oxidative stress are usually associated with chemical toxicity, and the stimulation by exogenous substances induces intracellular ROS accumulation and damage to the organisms [34]. In the process of ROS accumulation, organisms use SOD, CAT, and GSH to eliminate excess ROS to maintain dynamic homeostasis [35]. In this study, we found that N, N-dimethylaniline exposure resulted in a significant increase in ROS in the heart region of zebrafish embryos and decreased SOD and CAT activities as well as GSH levels. SOD and CAT, as antioxidants for first-line defense, dismutate superoxide radicals and breakdown hydrogen peroxides and hydroperoxides to harmless molecules, respectively [36]. GSH is the most essential antioxidant, scavenging oxidants and preventing oxidative stress, maintaining redox homeostasis in cells [37]. In our research, high levels of ROS may ultimately lead to reduced antioxidant enzyme activity and excessive GSH consumption, resulting in the accumulation of ROS in zebrafish embryos. MDA is the end product of lipid peroxidation, and when free radicals are increased, MDA levels are also elevated [38]. MDA is commonly used to evaluate the degree of oxidative damage in organisms [39]. The increase in MDA levels in our study further confirms the oxidative damage caused by the accumulation of ROS in zebrafish embryos.

Previous studies have shown that oxidative stress may induce abnormal lipid metabolism and apoptosis [40,41]. In this study, lipid accumulation and apoptosis were observed in the cardiac region of zebrafish embryos after N, N-dimethylaniline exposure, and these phenomena were positively correlated with the exposure concentration. Oxidative stress may be the potential mechanism for these pathological changes. In conclusion, these results indicate that N, N-dimethylaniline induced oxidative stress in zebrafish, leading to abnormal lipid metabolism and apoptosis.

The heart is the first organ to develop in the embryos and begins to function by the third day of embryo development in zebrafish, though it is not yet fully mature [42]. The developmental process of the heart is highly sensitive to environmental pollutants [43]. In our study, we counted cardiac function indices such as pericardial area, SV-BA spacing, heart rate, and RBC flow rate [44]. The results showed that N, N-dimethylaniline significantly increased the pericardial area and SV-BA spacing and significantly decreased the heart rate and RBC flow rate. The results of H&E sections and the average fluorescence intensity of cardiomyocytes showed that N, N-dimethylaniline significantly affected the morphologic structure of the heart, resulting in a reduction in the number of cardiomyocytes and a decrease in the thickness of the ventricle wall. These results suggest that N, N-dimethylaniline may have adverse effects on the normal development and function of the heart.

In addition, this study examined the expression levels of genes related to cardiac development and function, which usually play crucial roles in cardiac morphogenesis, cardiomyocyte proliferation and differentiation, and the maintenance of cardiac function. *myl7* is widely expressed throughout cardiac tissue, *vmhc* is specifically expressed in the ventricle, and *myh6* is specifically expressed in the atrium [45,46,47]. The downregulation of the expression of all three genes indicates that N, N-dimethylaniline interferes with the cardiac development process, leading to morphological and functional developmental defects of the atrium and ventricle. Following exposure to N, N-dimethylaniline, the observed phenomena of decreased heart rate and RBC flow rate may indicate impaired cardiac contractile function. Based on this observation, we hypothesized that these changes in cardiac function may be related to atrioventricular valve dysplasia. We therefore examined genes associated with atrioventricular valve development. The *bmp4* gene plays a crucial role in the formation of cardiac atrioventricular valves and can influence cellular differentiation and tissue morphology of the cardiac valves through its signaling pathway [48]. The expression of *tbx2b* is essential for the formation of cardiac jelly, and by regulating the expression of genes associated with the synthesis of cardiac jelly, it plays a key role in the formation and maturation of cardiac valves [49]. N, N-dimethylaniline induces the downregulation of both, with potential effects on heart valve development in zebrafish embryos. In addition, we analyzed molecular markers of atrioventricular valve precursors to explore at the molecular level how N, N-dimethylaniline affects cardiac valve development. The gene *has2* plays an important role in the formation of cardiac jelly and the transformation of epithelial cells into mesenchymal stroma, giving an important material basis for valve development [50]. The downregulation of the *has2* gene indicates that it has potential effects on heart valve development in zebrafish embryos. However, this hypothesis needs more experimental data to be further tested. In conclusion, we hypothesize that the heart may be the potential target for the toxic effects of N, N-dimethylaniline.

## 5. Conclusions

In this study, the significant effects of N, N-dimethylaniline on heart development were revealed through the use of a zebrafish embryo model, suggesting that the heart may be the main target organ for its toxic effects. This finding not only provides an important scientific basis for the environmental risk assessment of N, N-dimethylaniline but also highlights its potential threat to the health of aquatic organisms. In addition, future studies could focus on specific cardiac developmental markers and the assessment of cardiac function under longer exposure to further validate our hypothesis.

## Figures and Tables

**Table 1:** The primer sequences for qRT-PCR.

Gene	Forward (5′-3′)	Reverse (5′-3′)
*β-actin*	TGGACTCTGGTGATGGTGTGAC	GAGGAAGAAGAGGCAGCGGTTC
*myl7*	CCAAGAGGGGGAAAACTGCT	CGGTTCTGATCTATGCAGCCA
*vmhc*	CAGTGAGGCGGTGAAAGGAA	TCTGCAGCCCTCTTGTAAGC
*myh6*	GCTCCTTCCTCGGTGTGAAA	TTTTCAGACTCGGCGCTCTT
*bmp4*	AAGATCCACCAGTCGAGCCA	AGTTCGTCCTCTGGGATGCT
*tbx2b*	CGGAGATGCCAAAGCGAATG	ACAATATGGAACCTGGGCTGA
*has2*	CCTGGAGGACTGGTATGATC	CACACAATGCTAACACAACCAC

## Data Availability

Dataset available on request from the authors.
